# The Hospital Mortuary

**Published:** 1912-08-31

**Authors:** Margaret L. Tyler

**Affiliations:** Linden House, Highgate Road, N.W.


*To the Editor of* The Hospital.

### The Hospital Mortuary.

*To the Editor of* The Hospital.

Sir,—I read with much interest your remarks about mortuary arrangements at hospitals in a recent issue of The Hospital, and think the account of our new mortuary and pathology department at the London Homœopathic Hospital may interest you. You will see how the feelings of the bereaved relations are considered, and you would be touched and charmed if you would visit our little viewing chapel. If you cared to do so, we are always delighted to show the hospital, of which we are so proud. Mr. Attwood, the secretary, would welcome you; or on a Tuesday, Wednesday, or Thursday afternoon you would find me there, pleased to take you round.—Yours truly,

Margaret L. Tyler, M.D.

Linden House, Highgate Road, N.W., August 26.

[The account of the new mortuary to which Dr. Tyler refers is as follows :—"The extension of the pathological department, by taking in the old mortuary and viewing chapel, necessitated the building of a new mortuary, post-mortem room, and viewing chapel. This has been carried out at a cost of £800. The viewing chapel has been universally admired for its internal beauty, and Lady Tyler has added to her generosity by contributing handsome stained-glass windows, executed by Mr. Archibald K. Nicholson. . . It has always been the rule at this hospital, and one most willingly carried out, that, instead of the porter, the sister or nurse who has nursed the patient should take the friends to see their dead; this ensures that nothing is omitted in the matter of reverence, care, and sympathy, as well as due consideration for the feelings of the mourners." We shall hope at a convenient date to visit the new mortuary, and thank Dr. Tyler for her invitation.—Ed. The Hospital.]

